# Mental disorders: exploring normality models to distinguish what is normal from what is illness

**DOI:** 10.1192/j.eurpsy.2024.1370

**Published:** 2024-08-27

**Authors:** A. Fernandes, M. Gomes

**Affiliations:** ^1^Hospital de Braga, Braga, Portugal; ^2^Psychiatry Department, Hospital de Braga, Braga, Portugal

## Abstract

**Introduction:**

When reading about psychopathology what we find described are experiences similar to our own. Psychiatry deals with anguish, fear, motivation, choice, and many other aspects that makes us human. However, even though psychopathology is rooted in common human experience, mental disorders are often outside the experience of those who don’t suffer from it. Therefore, the distinction between normality and disease is central to psychiatry. The DSM proposes that mental disorders are necessarily linked to distress and/or impairment. However, it adds that the syndrome or pattern must not be an expectable response to an event - it excludes “normal” experiences and responses from the realm of mental illness. But how do we distinguish normal distress from illness? This review investigates how different meanings of normality can help us discern the fine line between mental illness and ordinary human experience.

**Objectives:**

We intend to critically examine and compare different models of normality. Additionally, we seek to discern the implications of these models for distinguishing mental disorders from normal mental experiences.

**Methods:**

Review of the literature.

**Results:**

We analyzed definitions and models of normality throughout the literature and selected the most relevant ones according to their popularity and/or strength of argument. Different models of normality (e.g. Biostatistical, Process, Health, Ideal, Biological advantage, etc.) were examined and compared, and the conceptualization of mental disorder was examined through the lens of each of these frameworks. Our investigation reveals the multifaceted nature of normality, with different models offering unique perspectives on mental health. From statistical approaches to cultural considerations, each model contributes distinct criteria for distinguishing what is normal from what is illness. By synthesizing these results, we gain a comprehensive view of the factors influencing the conceptualization of normality in the context of mental health.

**Conclusions:**

This review emphasizes the importance of adopting a nuanced, cautious and multifactorial approach when discerning mental disorders from normal experiences. Rather than relying on a singular definition, our analysis suggests that a comprehensive understanding of normality can help us to better discern what is normal and what is illness.

**Disclosure of Interest:**

None Declared

